# Supplementary material for: β2-adrenergic receptor regulates ER-mitochondria contacts
Source: Sci Rep. 2021 Nov 2;11:21477. doi: 10.1038/s41598-021-00801-w (PMC8563895; doi:10.1038/s41598-021-00801-w)
Supplement: Supplementary file 1 — Supplementary Information. [file 41598_2021_801_MOESM1_ESM.pdf]

Supplementary Information for

β2-adrenergic receptor regulates ER-mitochondria contacts

Youngshin Lim[a, b, 1], Il-Taeg Cho[a, c, 1], Helmut G. Rennke[a], and Ginam Cho[a, b *]

[a] Department of Pathology, Brigham and Women's Hospital and Harvard Medical School, Boston, MA 02115, USA.

[b] Current address: Department of Pathology and Laboratory Medicine, Cedars-Sinai Medical Center, Los Angeles, CA 90048, USA

[c] Current address: Department of Surgery, Keck School of Medicine, University of Southern California, Los Angeles, CA 90033, USA.

[1] Equal contribution

* Correspondence: ginam.cho@cshs.org

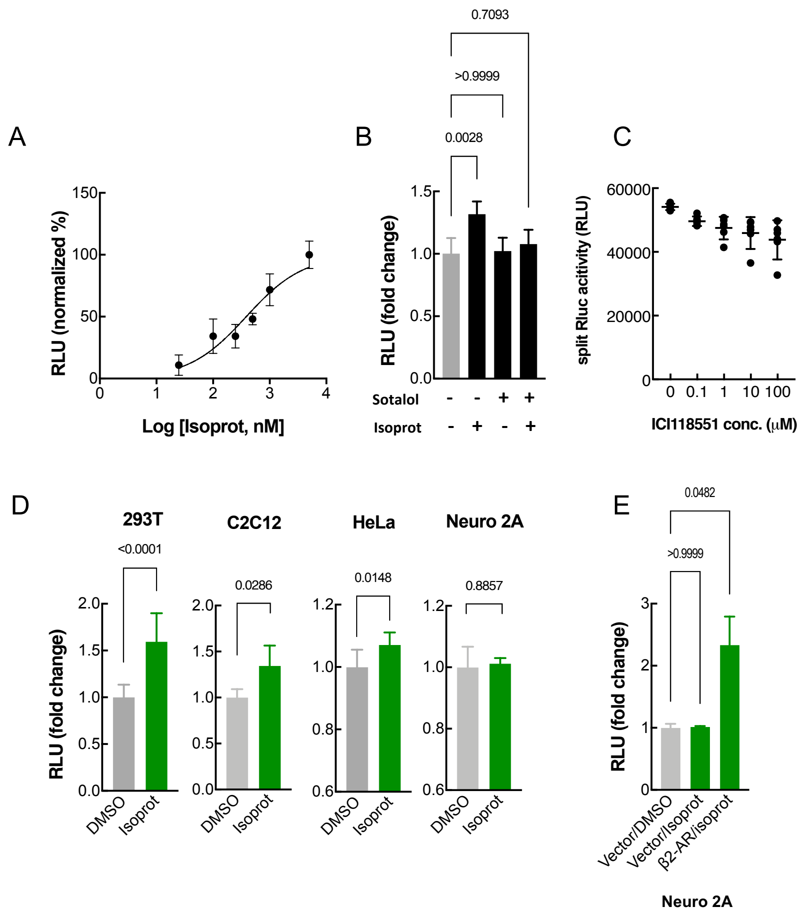

Figure S1. ***A,*** A concentration-responsive curve of isoproterenol (25, 100, 250, 500, 1000, 5000 nM) effect on split-Rluc activity (*n* = 16; nonlinear regression (non-linear fit, EC50 = 385.4 nM); Error bars: SEM. ***B,*** The effect of β-AR antagonist (sotalol, 250 μM) on isoproterenol (isoprot)-induced split-Rluc activity. *n = 6*; Kruskal-Wallis test with Dunn's multiple comparisons test. ***C,*** Split-Rluc activities (RLU, relative light unit) of HEK293T cells treated with different concentrations of ICI 118551 hydrochloride, an inverse β2-AR agonist. ***D,*** Split-Rluc activities (fold change, normalized with the mean of DMSO) of HEK293T, HeLa, C2C12 or Neuro2a cells, treated with DMSO (control) or isoproterenol (1 μM). *n* = 15 (293T), 4 (C2C12 and N2a), 8 (HeLa); Unpaired two-tailed t-test (for 293T); Mann-Whitney test (C2C12, HeLa, N2a). 293T data presented here are from the same set data used in Fig 2SA, just for easy comparison. ***E,*** Split Rluc activities of the Neuro2a cells transfected with vector or β2-AR construct treated with DMSO or isoprot as indicated. All data in B, D,E: mean ± SD; data in C: median with interquartile range. *p* values (if ≤ 0.05, significant) are indicated at the top of each graph.

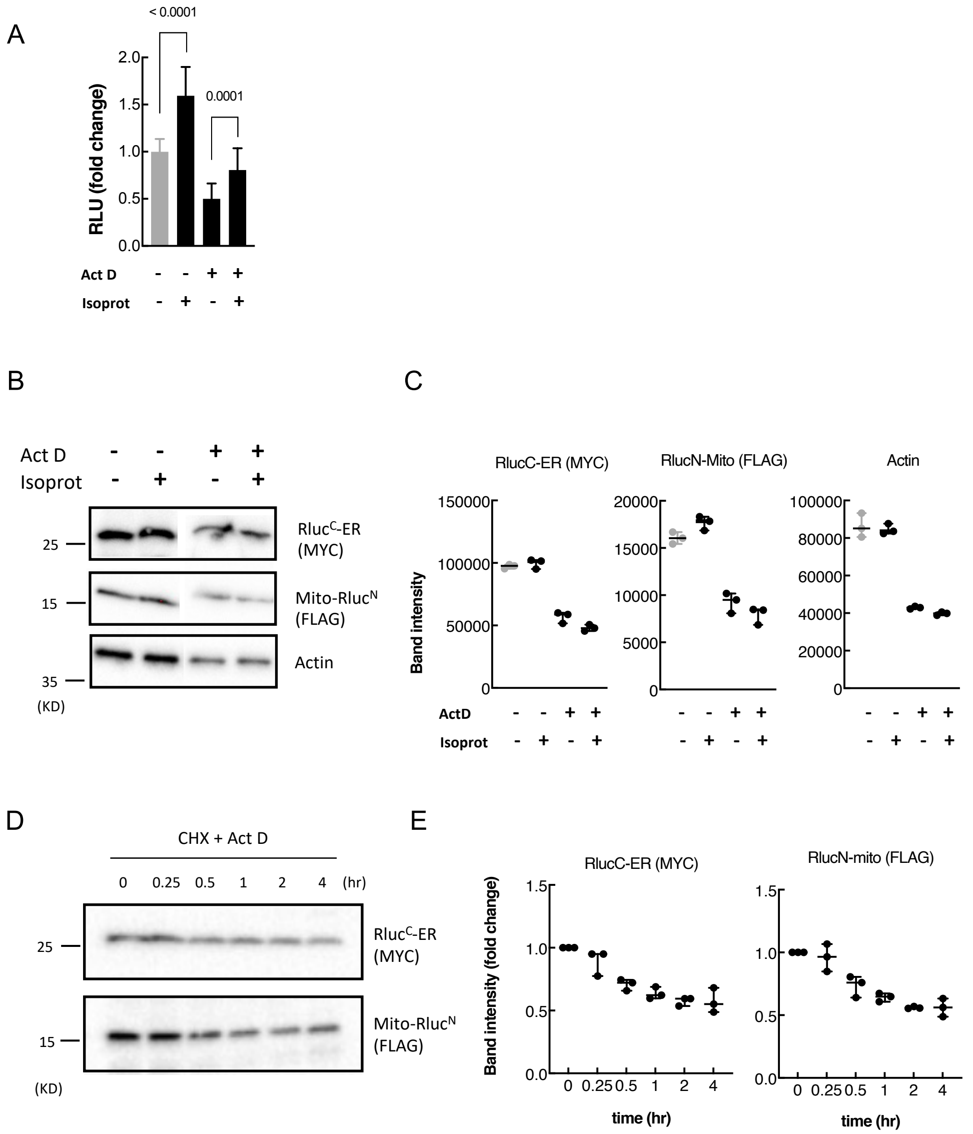

Figure S2. ***A,*** The effect of the transcription inhibitor actinomycin D (ActD, 0.8 μM) on isoproterenol (1 μM)-induced split-Rluc activity. One-way ANOVA with Sidak's multiple comparisons test (*n* = 15). ***B,*** The effect of Act D and isoproterenol on the expression level of split-Rluc fragments. Western blot analysis of split-Rluc fragments in HEK293T cells treated with indicated drugs, probed with MYC (for $Rluc^{C}$-ER), FLAG (for Mito-$Rluc^{N}$), or actin (loading control) antibody. Full-length blots are presented in Supplementary Figure 7. ***C,*** Quantification of Western blot analysis as shown in C. ***D,*** Western blot analysis of protein levels for split-Rluc fragments in HEK293T cells treated with Act D (0.8 μM) and cycloheximide (protein translation inhibitor, 20 μg/ml). Samples were taken at the time points indicated and probed with MYC or FLAG antibody. Full-length blots are presented in Supplementary Figure 8. ***E,*** Quantification of Western blot analysis as shown in D. Data in A: mean ± SD; data in C, E: median with interquartile range. *p* values (if ≤ 0.05, significant) are indicated at the top of each graph.

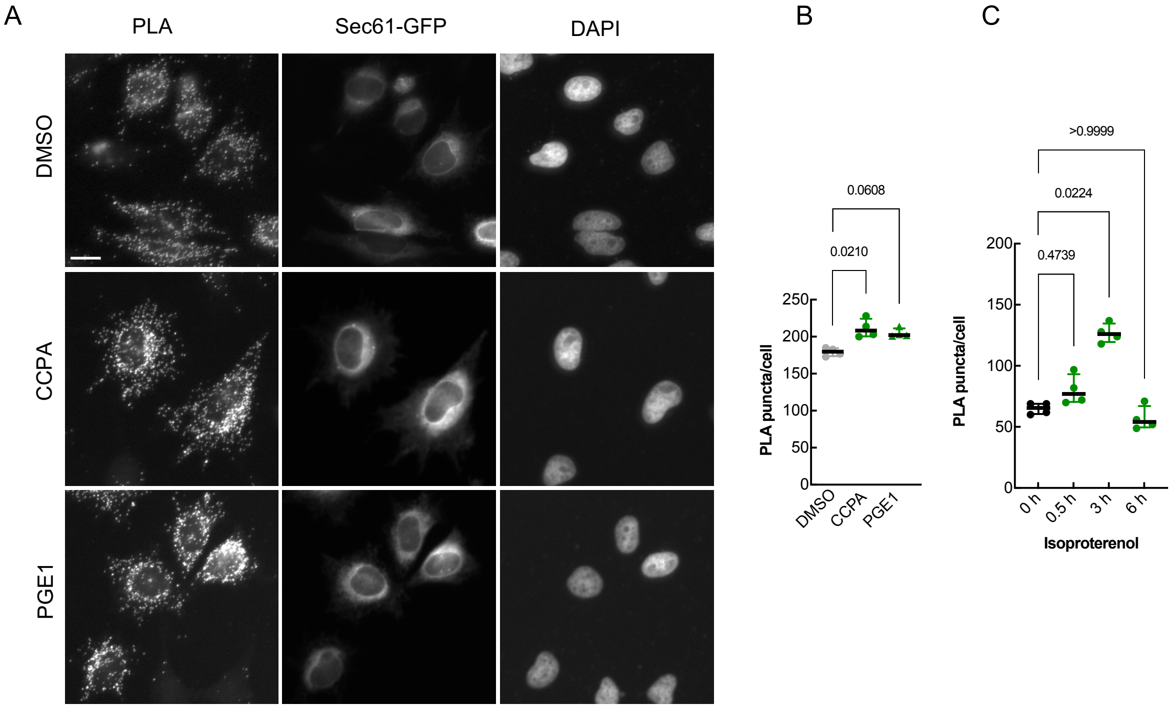

Figure S3. ***A,*** Representative images of proximity ligation assay (PLA) in HeLa cells treated with DMSO, CCPA, or PGE1 (1 μM each). PLA fluorescent signal indicates a close apposition between ER and Mito. Sec61-GFP, to label ER; DAPI, nucleus. ***B,*** Quantifications of PLA signals as shown in A. Kruskal-Wallis test with Dunn's multiple comparisons test (*n* = 4 with 77-97 cells each). ***C,*** Quantification of PLA in HeLa cells treated with isoproterenol (1 μM) for 0 (DMSO), 0.5, 3, or 6 hours (*n* = 4 with 71, 52, 48, 56 cells, respectively; Kruskal-Wallis test with Dunn's multiple comparison test). ***C,*** Representative images of TMRM (measuring mitochondrial membrane potential) fluorescent signal in HeLa cells treated with DMSO or CCPA (1 μM each). *p* values (if ≤ 0.05, significant) are indicated at the top of each graph. All data: median with interquartile range. Scale bars: 10 μm

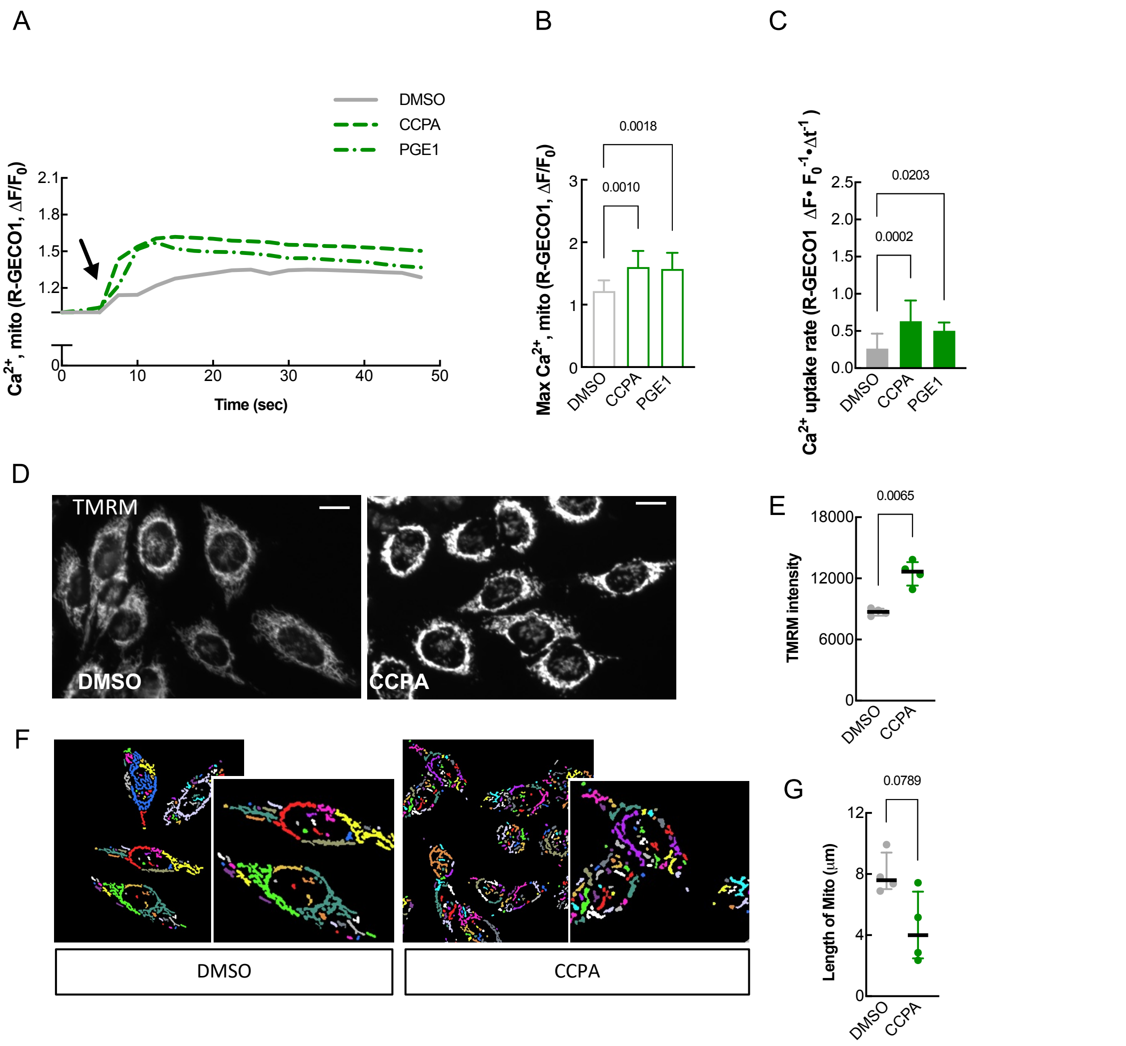

Figure S4. ***A,*** Mito Ca^{2+} uptake in response to histamine (100 μM). Mito-R-GECO1 fluorescent intensity change averaged over multiple cells (HeLa) is plotted every 2.5 sec in DMSO-, CCPA-, or PGE1- treated HeLa cells. Arrow: histamine addition. ***B,*** Maximum peak of Mito Ca^{2+} uptake (as in A). One-way ANOVA with Dunn's multiple comparisons test (n = 10, 13, 15 for DMOS, CCPA, PGE1). ***C,*** Mito Ca^{2+} uptake rate (as in A; 7.5 to 12.5 sec). Kruskal-Wallis test with Dunn's multiple comparisons test (n = 12, DMSO; 15, CCPA and PGE1). A-C are from the sister experiments shown in Fig 2A-C. ***D,*** Representative images of TMRM signal (for Mito membrane potential) (HeLa cells). ***E,*** Quantification of TMRM intensity as in E. Unpaired two-tailed *t*-test with Welch's correction (n = 4 with 52 cells each). ***F,*** Representative images of Mito (converted in Image Analyst MKII software) used for length comparison (HeLa cells, 1 μM drug). ***G,*** Quantification of Mito length as in F. Mann Whitney test ($n = 4$ with 278 (DMSO) or 425-445 (CCPA) Mito for each experiment). Each drug: used at 1 μM final conc. Data in B, C: mean ± SD; data in E, G: median with interquartile range. Scale bars: 10 μm.

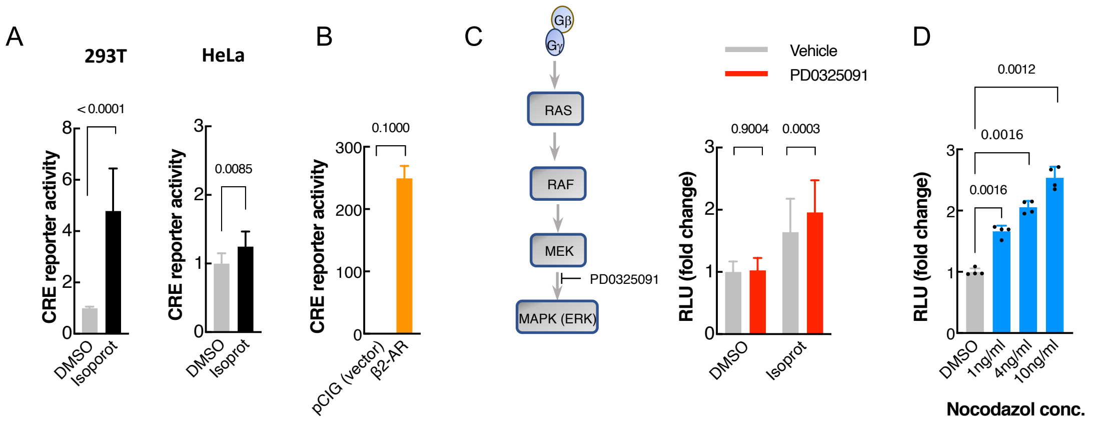

Figure S5. ***A,*** cAMP response element (CRE) reporter gene assay in HEK293T (A) or HeLa (B) cells treated with DMSO or isoproterenol (isoprot, 1 μM). *n* = 9; Two-tailed unpaired *t*-test. ***B***, cAMP response element (CRE) reporter gene activity in HEK293T cells transfected with pCIG (vector expressing GFP) or β2-AR expression construct. *n* = 3; Mann Whitney test. ***C,*** (Left) Simplified schematic depicting $G_{\beta\chi}$ downstream pathway leading to activation of MAPK. PD0325091 is a chemical inhibitor of MAPK. (Right) Split-Rluc activities (RLU, normalized to the mean of DMSO/Vehicle treated) of the HEK293T cells treated with vehicle or PD0325091 (1 μM, MAPK inhibitor), in the presence of DMSO or isoproterenol (1 μM). *n* = 20. 2-way ANOVA with Sidak's multiple comparisons test. ***D,*** Split-Rluc activities (RLU, normalized to the mean of DMSO) of the HEK293T cells treated with DMSO or indicated concentrations of nocodazole. *n* = 4. 1-way ANOVA with Dunnett's multiple comparisons test. *p* values (if ≤ 0.05, significant) are indicated at the top of each graph. All data: mean ± SD.

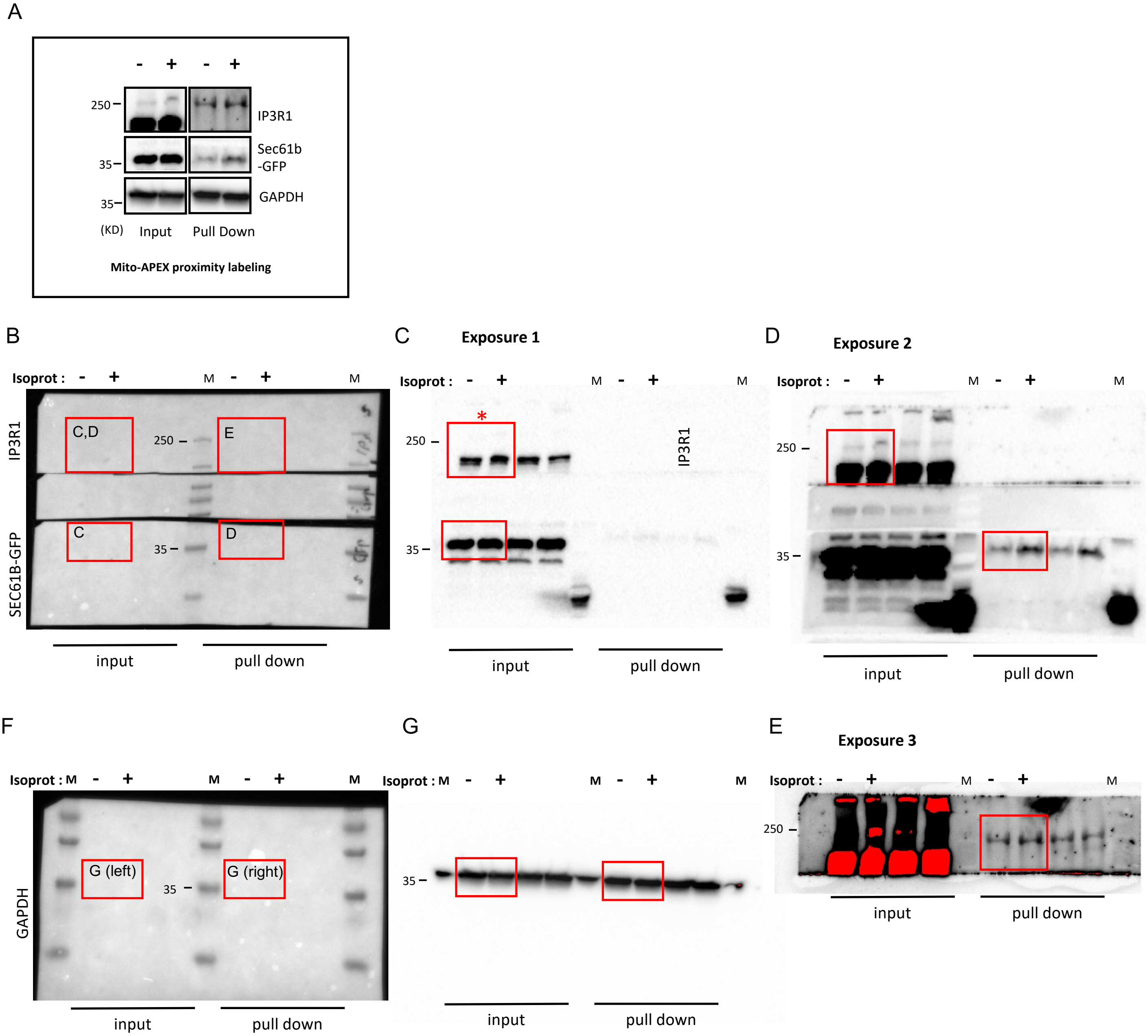

Figure S6. ***A,*** Cropped images of the Western blot data presented in Fig. 2G. ***B***, Uncropped image of the original blot cut into 3 different pieces used for IP3R or Sec61bGFP detection in A. Each red box labeled with C, D, E indicates each corresponding boxed area in C, D, and E. ***C-E,*** Uncropped images of the developed blots probed for IP3R1- or GFP (Sec61bGFP). * in C indicates the corresponding cropped area in D but with a shorter exposure. Blot in E is with enhanced development (Red highlighted bands: overexposed ones). ***F,*** Image of the original blot used for GAPDH detection in A. Red boxes labeled with G left or G right indicates the corresponding areas shown in G. ***G,*** Uncropped images of the developed Western blot probed for GAPDH. M: molecular weight size marker (numbers are in kD).

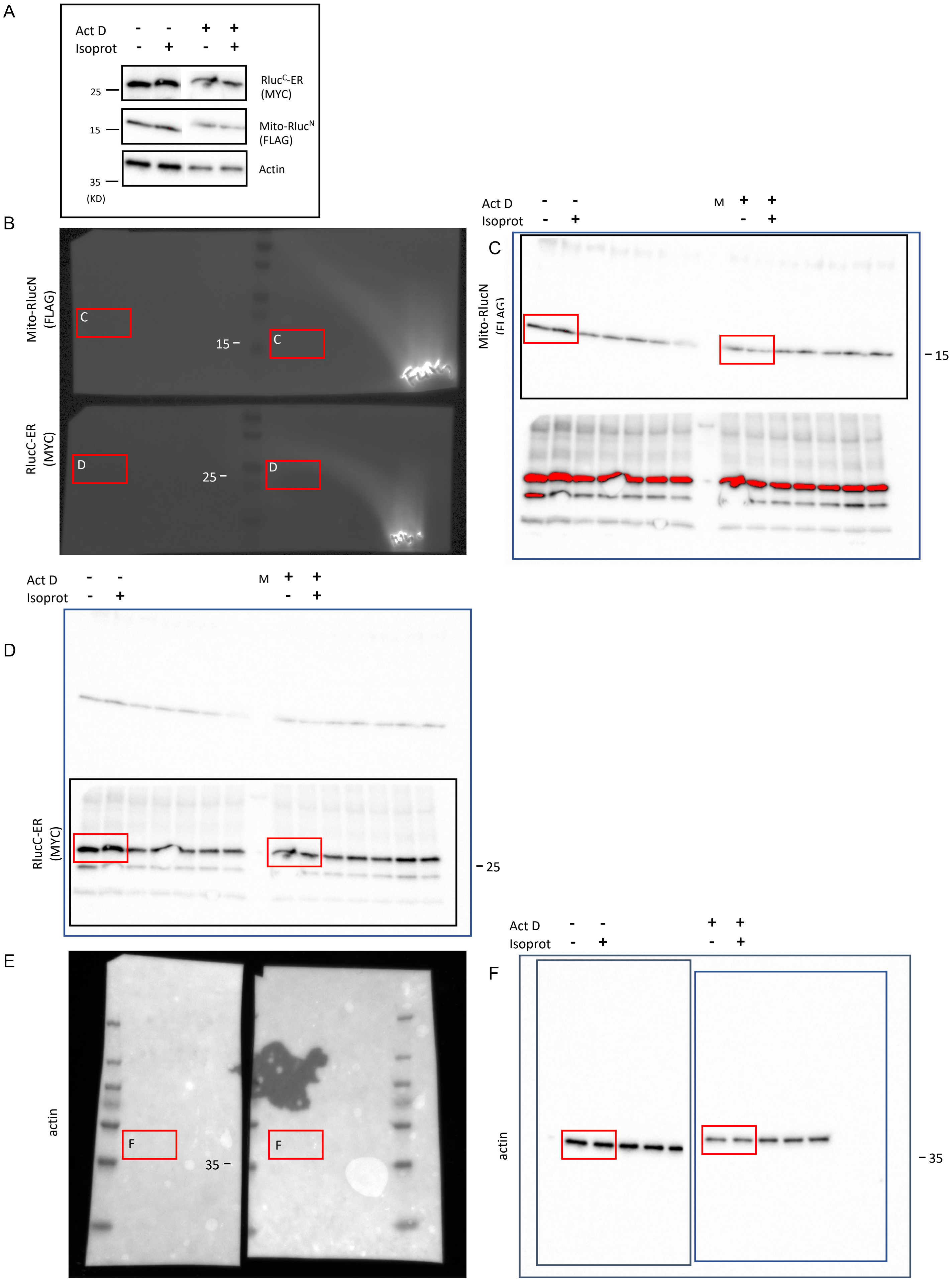
A
Act D - - + +
Isoprot - + - +
25
15
35
(KD)
Rluc^{C}-ER (MYC)
Mito-Rluc^{N} (FLAG)
Actin
B
Mito-RlucN (FLAG)
RlucC-ER (MYC)
15
25
C
D
Act D - - M + +
Isoprot - + - +
Mito-RlucN (FLAG)
15
Act D - - M + +
Isoprot - + - +
RlucC-ER (MYC)
25
E
actin
35
F
Act D - - + +
Isoprot - + - +
actin
35

Figure S7. ***A,*** Western blot data (cropped images) presented in Fig. S2B. ***B,*** Uncropped image of the original blots used for FLAG (top, for Mito-Rluc^{N}) and MYC (bottom, for Rluc^{C}-ER) detection shown in A. Red boxes labeled with C and D indicate the corresponding areas in C and D. ***C, D,*** Uncropped images of the developed blots probed for FLAG (C, for Mito-Rluc^{N}) or MYC (D, for Rluc^{C}-ER). B and C are the same blots with different exposure time. ***E,*** Uncropped image of the original blots used for actin detection shown in A. Red boxes labeled with F indicate the corresponding areas in F. **F,** Uncropped image of the developed blots probed for actin. Of note, the blots were blocked with 1% skim milk. M: molecular weight size marker (numbers are in kD). Red boxes indicate the cropped areas used in A (also Fig S2B).

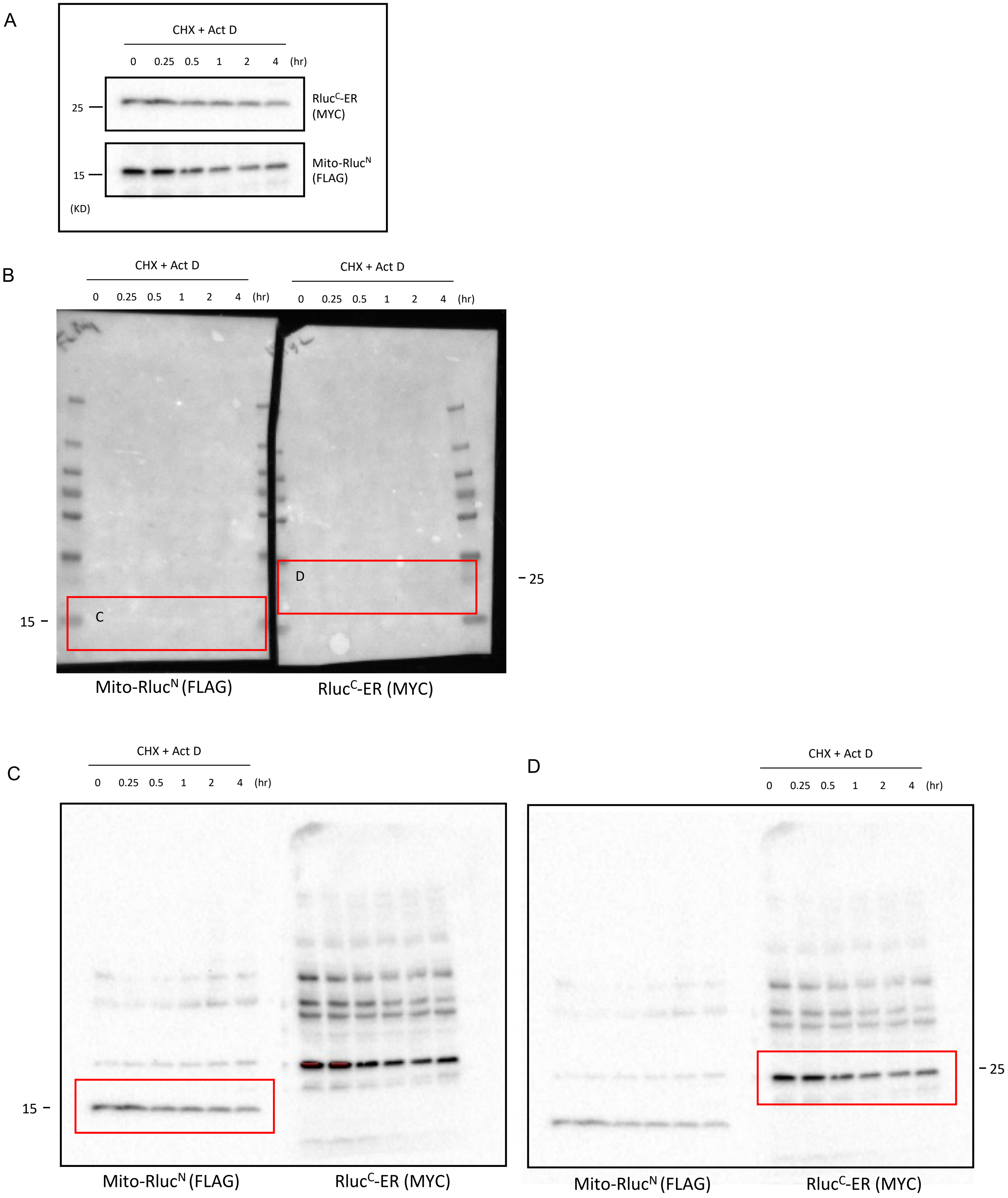

Figure S8. ***A,*** Western blot data (cropped) presented in Fig. S2C. ***B,*** Uncropped image of the original blots used for FLAG (left, for Mito-Rluc^N) and MYC (right, for Rluc^C-ER) detection shown in A. Red boxes labeled with C and D indicate the corresponding boxed areas in C and D. ***C-D,*** Uncropped images of the developed blots probed for FLAG (C, for Mito-Rluc^N) and for MYC (D, for Rluc^C-ER). C and D: the same blot with a different exposure time. Of note, blots were blocked with 1% BSA. M: molecular weight size marker (numbers are in kD). Red boxes indicate the cropped areas used in A (also Fig S2C).

Table S1. Potential hits from the initial screen

NCC_STRUC_ID	Drug Name	Target Pathway	Target GPCR	Target AR	Agonist/ Antagonist	sRluc activity
CPD000471620	Formoterol	GPCR	Adrenergic Rc	beta-2	agonist	81826.7
CPD000466295	Salmeterol	GPCR	Adrenergic Rc	beta-2	agonist	81826.7
CPD000058267	Isoproterenol	GPCR	Adrenergic Rc	beta	agonist	81826.7
CPD000857209	Epinephrine	GPCR	Adrenergic Rc	alpha, beta	agonist	81826.7
CPD000058335	Triamcinolone (76-25-	Glucocorticoid Rc				81826.7
CPD000058729	Duvadilan	GPCR	Adrenergic Rc	beta	agonist	45628.9
CPD001453712	Metaproterenol	GPCR	Adrenergic Rc	beta	agonist	45628.9
CPD000058329	Fluocinolone	Glucocorticoid Rc				45628.9
CPD000466308	Epirubicin	DNA Related				45628.9
CPD000469221	Balsalazide	GPCR	Prostaglandin Rc			45628.9
CPD000112594	Prostaglandin E1	GPCR	Prostaglandin Rc			45628.9
CPD001496939	Terbutaline	GPCR	Adrenergic Rc	beta-2	agonist	39806.7
CPD000466344	Topotecan	DNA Related				39806.7
CPD000466294	RU 24969	GPCR	Serotonergic Rc			39806.7
CPD000468732	CCPA	GPCR	Adenosine Rc			39806.7
CPD000058515	Artane	GPCR	mAChR			39806.7
CPD001491672	Phylloquinone	Vitamine K1				39806.7
CPD000058513	Salbutamol	GPCR	Adrenergic Rc	beta-2	agonist	33662.2
CPD000058420	Betaxolol	GPCR	Adrenergic Rc	beta-1	antagonist	33662.2
CPD000058383	Norepinephrine	GPCR	Adrenergic Rc	alpha, beta	agonist	33662.2
CPD000466920	Beclomethasone	Glucocorticoid Rc				33662.2
CPD000058570	Doxorubicin	DNA Related				33662.2
CPD000466296	SB 205607	GPCR	Opioid Rc			33662.2
CPD000035778	Hydrochlorothiazide	Transporter				33662.2
CPD000499581	Valproic acid	HDAC				33662.2
CPD000058745	Clobetasol	Glucocorticoid Rc				19273.3
CPD001491664	Amcinonide	Glucocorticoid Rc				19273.3
CPD000449294	Capsaicin	Vanilloid Rc				19273.3
CPD000466355	Idarubicin	DNA Related				19273.3
CPD000466319	Lamivudine	DNA Related				19273.3
CPD000468734	PD 81723	GPCR	Adenosine Rc			19273.3
CPD000469232	Lofexidine	GPCR	Adrenergic Rc	alpha-2	agonist	19273.3

Table S2. GPCR-associated drugs (in NCC library)

NCC_Structure_ID	Target GPCR	sRluc activity
CPD000058832	Acetylcholine	14504.4
CPD000058661	Acetylcholine	14504.4
CPD000058821	Acetylcholine	14084.4
CPD000449327	Acetylcholine	13964.4
CPD000469231	Acetylcholine	11964.4
CPD000449286	Acetylcholine	11773.3
CPD001906769	Acetylcholine	11711.1
CPD000058719	Acetylcholine	11673.3
CPD000449292	Acetylcholine	11620.0
CPD000058660	Acetylcholine	11517.8
CPD000471626	Acetylcholine	11308.9
CPD000058672	Acetylcholine	11186.7
CPD000469196	Acetylcholine	11055.6
CPD000058605	Acetylcholine	10964.4
CPD000469284	Acetylcholine	10846.7
CPD000058817	Acetylcholine	10846.7
CPD001906775	Acetylcholine	10647.8
CPD000059171	Acetylcholine	10613.3
CPD000449267	Acetylcholine	10588.9
CPD000059182	Acetylcholine	10124.4
CPD000466270	Acetylcholine	10033.3
CPD000469282	Acetylcholine	5960.0
CPD000057879	Acetylcholine	8077.8
CPD000058515	Acetylcholine	39806.7
CPD000058523	Acetylcholine	13266.7
CPD000471622	Acetylcholine	13113.3
CPD000059142	Acetylcholine	12557.8
CPD000059053	Acetylcholine	12497.8
CPD000058490	Acetylcholine	11915.6
CPD000058680	Acetylcholine	11593.3
CPD001906768	Acetylcholine	11055.6
CPD000058623	Acetylcholine	10682.2
CPD000466280	Acetylcholine	10601.1
CPD000058440	Acetylcholine	10257.8
CPD001496929	Acetylcholine	9533.3
CPD000058572	Acetylcholine	15577.8
CPD000449277	Acetylcholine	12695.6
CPD000471625	Acetylcholine	12031.1
CPD000875213	Acetylcholine	11786.7
CPD000058736	Acetylcholine	12324.4
CPD000468732	Adenosine	39806.7
CPD000468734	Adenosine	19273.3

CPD000048468	Adenosine	12986.7
CPD000466321	Adenosine	11606.7
CPD000466392	Adenosine	10846.7
CPD000449316	Adenosine	10733.3
CPD000058553	Adenosine	10555.6
CPD000449284	Adenosine	10148.9
CPD000469631	Adenosine	17306.7
CPD000466364	Adenosine	15860.0
CPD000469168	Adenosine	14800.0
CPD000471620	Adrenergic	81826.7
CPD000466295	Adrenergic	81826.7
CPD000058267	Adrenergic	81826.7
CPD000857209	Adrenergic	81826.7
CPD000058729	Adrenergic	45628.9
CPD001453712	Adrenergic	45628.9
CPD001496939	Adrenergic	39806.7
CPD000058513	Adrenergic	33662.2
CPD000058420	Adrenergic	16197.8
CPD000058383	Adrenergic	33662.2
CPD000469232	Adrenergic	19273.3
CPD000058800	Adrenergic	16197.8
CPD000058463	Adrenergic	15577.8
CPD000499584	Adrenergic	14606.7
CPD000469190	Adrenergic	14555.6
CPD000058525	Adrenergic	14412.2
CPD000466362	Adrenergic	14320.0
CPD000058423	Adrenergic	14320.0
CPD000059167	Adrenergic	14320.0
CPD000148117	Adrenergic	14146.7
CPD000449302	Adrenergic	14146.7
CPD000449280	Adrenergic	13964.4
CPD000466276	Adrenergic	13964.4
CPD000059120	Adrenergic	13964.4
CPD000466346	Adrenergic	13822.2
CPD000466347	Adrenergic	13660.0
CPD000472527	Adrenergic	13528.9
CPD000326828	Adrenergic	13222.2
CPD000550486	Adrenergic	13222.2
CPD000058388	Adrenergic	13148.9
CPD000449301	Adrenergic	12813.3
CPD000449266	Adrenergic	12733.3
CPD001370753	Adrenergic	12695.6
CPD001819784	Adrenergic	12466.7
CPD000466340	Adrenergic	12324.4
CPD000059081	Adrenergic	12310.0

CPD000058833	Adrenergic	12222.2
CPD000058355	Adrenergic	12160.0
CPD000449297	Adrenergic	12095.6
CPD000036768	Adrenergic	12047.8
CPD000058295	Adrenergic	12031.1
CPD000449270	Adrenergic	12020.0
CPD000059111	Adrenergic	11940.0
CPD000149600	Adrenergic	11915.6
CPD000058500	Adrenergic	11860.0
CPD001906782	Adrenergic	11860.0
CPD000058368	Adrenergic	11830.0
CPD000238156	Adrenergic	11711.1
CPD000469292	Adrenergic	11673.3
CPD000058230	Adrenergic	11555.6
CPD000469209	Adrenergic	11453.3
CPD000036827	Adrenergic	11453.3
CPD000449282	Adrenergic	11333.3
CPD001453705	Adrenergic	11308.9
CPD000097306	Adrenergic	11308.9
CPD001496977	Adrenergic	11240.0
CPD000059074	Adrenergic	11055.6
CPD000469177	Adrenergic	10942.2
CPD000059133	Adrenergic	10914.4
CPD000471619	Adrenergic	10846.7
CPD000058803	Adrenergic	10846.7
CPD000466275	Adrenergic	10780.0
CPD000058975	Adrenergic	10780.0
CPD000058416	Adrenergic	10780.0
CPD000059045	Adrenergic	10588.9
CPD000449268	Adrenergic	10511.1
CPD000058180	Adrenergic	10424.4
CPD000449273	Adrenergic	10344.4
CPD000469141	Adrenergic	10257.8
CPD000058309	Adrenergic	10148.9
CPD000058486	Adrenergic	10103.3
CPD000469154	Adrenergic	10082.2
CPD000058365	Adrenergic	10082.2
CPD000058422	Adrenergic	9828.9
CPD000058520	Adrenergic	9760.0
CPD000466345	Adrenergic	9731.1
CPD000326936	Adrenergic	9731.1
CPD000471623	Adrenergic	9591.1
CPD000058296	Adrenergic	9591.1
CPD000469136	Adrenergic	9533.3
CPD000058219	Adrenergic	9533.3

CPD000058292	Adrenergic	8517.8
CPD000472526	Adrenergic	8077.8
CPD000466386	Angiotensin	9533.3
CPD000466359	Angiotensin	14606.7
CPD000059061	Angiotensin	12617.8
CPD000469199	Angiotensin	12310.0
CPD000466318	Angiotensin	11964.4
CPD001906784	Angiotensin	11711.1
CPD000469593	Angiotensin	11593.3
CPD000466326	Angiotensin	10986.7
CPD000499582	Angiotensin	9868.9
CPD000466337	Angiotensin	9648.9
CPD000466306	Angiotensin	8517.8
CPD000466284	Cannabinoid	13028.9
CPD000449274	Cannabinoid	12324.4
CPD000466379	Dopamine	14555.6
CPD000466274	Dopamine	13185.6
CPD000449309	Dopamine	12695.6
CPD000449276	Dopamine	12617.8
CPD000466292	Dopamine	12497.8
CPD000466366	Dopamine	12191.1
CPD000058471	Dopamine	12160.0
CPD000449283	Dopamine	12031.1
CPD000499578	Dopamine	11673.3
CPD000449275	Dopamine	11646.7
CPD001566944	Dopamine	10986.7
CPD000469142	Dopamine	10588.9
CPD000058504	Dopamine	10588.9
CPD000238142	Dopamine	10572.2
CPD000449298	Dopamine	10257.8
CPD000058411	Dopamine	9984.4
CPD001370746	Dopamine	9690.0
CPD000469143	Dopamine	14208.9
CPD000058470	Dopamine	11308.9
CPD000058957	Dopamine	12935.6
CPD000394012	Dopamine	13900.0
CPD000466293	Dopamine	10942.2
CPD000326935	Dopamine	15860.0
CPD000449303	Dopamine	12435.6
CPD000058186	Dopamine	11517.8
CPD000058380	Dopamine	11333.3
CPD000449328	Dopamine	10148.9
CPD000466383	Dopamine	10192.2
CPD000058465	Dopamine	10424.4
CPD000466323	Dopamine	13477.8

CPD000468736	Dopamine	11620.0
CPD000058450	GABA	13660.0
CPD000550478	GABA	13311.1
CPD000449279	GABA	12961.1
CPD000149316	GABA	12874.4
CPD000058398	GABA	12813.3
CPD001491654	GABA	12466.7
CPD000058418	GABA	12466.7
CPD000238177	GABA	12435.6
CPD000238180	GABA	12031.1
CPD000469176	GABA	12025.6
CPD000469160	GABA	11964.4
CPD000596519	GABA	11786.7
CPD000058501	GABA	11186.7
CPD000466325	GABA	10886.7
CPD000059151	GABA	10707.8
CPD000058302	GABA	10424.4
CPD000010931	GABA	10192.2
CPD000469145	GABA	9868.9
CPD000469226	GABA	6593.3
CPD000469289	GABA	6420.0
CPD000499581	GABA	33662.2
CPD000058410	GABA	18031.1
CPD000466378	GABA	15577.8
CPD000058433	GABA	14146.7
CPD000058855	GABA	10082.2
CPD000449307	GABA	11308.9
CPD000059075	GABA	10148.9
CPD000058464	GABA	10682.2
CPD000857229	GABA	18031.1
CPD000471616	Histamine	15860.0
CPD001370751	Histamine	14800.0
CPD000149358	Histamine	13964.4
CPD000058436	Histamine	13222.2
CPD000058462	Histamine	13131.1
CPD000059100	Histamine	12695.6
CPD000466384	Histamine	12617.8
CPD000469144	Histamine	12222.2
CPD001370748	Histamine	12222.2
CPD000469632	Histamine	12160.0
CPD001453715	Histamine	12095.6
CPD000466315	Histamine	11800.0
CPD000058353	Histamine	11773.3
CPD000653467	Histamine	11453.3
CPD000058255	Histamine	10846.7

CPD000058721	Histamine	10846.7
CPD000469183	Histamine	10445.6
CPD000058379	Histamine	9984.4
CPD000466271	Histamine	9788.9
CPD000469220	Histamine	9690.0
CPD000471617	Histamine	8755.6
CPD000718798	Histamine	8517.8
CPD000058961	Histamine	7140.0
CPD000469188	Leukotriene	14320.0
CPD000466377	Leukotriene	13477.8
CPD000466316	Leukotriene	13113.3
CPD000469147	Leukotriene	12064.4
CPD000058555	Leukotriene	10424.4
CPD000059165	Leukotriene	9868.9
CPD000466352	Leukotriene	9788.9
CPD000449311	NMDA	12047.8
CPD000449296	NMDA	11915.6
CPD000326694	NMDA	12095.6
CPD000058313	NMDA	10511.1
CPD000058445	NMDA	10445.6
CPD000058999	NMDA	10466.7
CPD000466296	Opioid	33662.2
CPD000449312	Opioid	13861.1
CPD000466389	Opioid	13822.2
CPD000058466	Opioid	13266.7
CPD000449308	Opioid	12222.2
CPD000449320	Opioid	11964.4
CPD000058908	Opioid	11593.3
CPD000469140	Opioid	10942.2
CPD000058766	Opioid	10124.4
CPD000449281	Opioid	8755.6
CPD000466297	Opioid	14800.0
CPD000058382	Platelets	10173.3
CPD000466348	Platelets	12733.3
CPD000112594	Prostanoid	45628.9
CPD000466354	Prostanoid	14800.0
CPD000042823	Prostanoid	8517.8
CPD000449318	Prostanoid	13861.1
CPD000718800	Prostanoid	13822.2
CPD000058785	Prostanoid	13528.9
CPD000469165	Prostanoid	13311.1
CPD000469178	Prostanoid	13148.9
CPD000058184	Prostanoid	13148.9
CPD000145728	Prostanoid	13113.3
CPD000466299	Prostanoid	12986.7

CPD000326718	Prostanoid	12813.3
CPD000466327	Prostanoid	12733.3
CPD000058991	Prostanoid	12047.8
CPD000040181	Prostanoid	12020.0
CPD000449291	Prostanoid	11860.0
CPD000058443	Prostanoid	11800.0
CPD000058746	Prostanoid	11692.2
CPD000059146	Prostanoid	11240.0
CPD000449290	Prostanoid	11186.7
CPD000058206	Prostanoid	11186.7
CPD000469285	Prostanoid	11108.9
CPD000469164	Prostanoid	10942.2
CPD000440694	Prostanoid	10733.3
CPD000058188	Prostanoid	10588.9
CPD000058461	Prostanoid	10344.4
CPD000469594	Prostanoid	10211.1
CPD000550473	Prostanoid	10082.2
CPD000058835	Prostanoid	9648.9
CPD000058286	Prostanoid	9271.1
CPD000466331	Prostanoid	8517.8
CPD000058723	Prostanoid	6593.3
CPD000058715	Prostanoid	6420.0
CPD000466294	Serotonin	39806.7
CPD000059115	Serotonin	5960.0
CPD000466283	Serotonin	18031.1
CPD000469200	Serotonin	15860.0
CPD000466268	Serotonin	13477.8
CPD000449310	Serotonin	13477.8
CPD000058254	Serotonin	13477.8
CPD001307702	Serotonin	13113.3
CPD000469228	Serotonin	12961.1
CPD000525252	Serotonin	12935.6
CPD000012114	Serotonin	12874.4
CPD000469138	Serotonin	12714.4
CPD000059131	Serotonin	12714.4
CPD000469203	Serotonin	12295.6
CPD000469191	Serotonin	12160.0
CPD000058507	Serotonin	12064.4
CPD000469211	Serotonin	11964.4
CPD000112269	Serotonin	11860.0
CPD000449305	Serotonin	11800.0
CPD000466269	Serotonin	11646.7
CPD000469179	Serotonin	11453.3
CPD000469156	Serotonin	11186.7
CPD000466298	Serotonin	11108.9

CPD000471618	Serotonin	11108.9
CPD001227191	Serotonin	11021.1
CPD000465669	Serotonin	10866.7
CPD000469233	Serotonin	10613.3
CPD000449271	Serotonin	10211.1
CPD000466277	Serotonin	10211.1
CPD000469158	Serotonin	10173.3
CPD000058431	Serotonin	9788.9
CPD000466272	Serotonin	9648.9
CPD000058452	Serotonin	7608.9
CPD000059105	Serotonin	6593.3
CPD000449269	Serotonin	4731.1
CPD000449272	Serotonin	12222.2
CPD000449287	Serotonin	11055.6
CPD000058269	Serotonin	6420.0
CPD001453706	Serotonin	10707.8
CPD000469181	Serotonin	10613.3
CPD000058926	Serotonin	12733.3
CPD000449299	Serotonin	13028.9

Table S3. AR-associated drugs (in NCC library)

NCC_STRUC_ID	Target AR	Agonist/ Antagonist	sRluc activity
CPD000469232	alpha	agonist	19273.3
CPD000058833	alpha	agonist	12222.2
CPD000059111	alpha	agonist	11940.0
CPD000058219	alpha	agonist	9533.3
CPD000058292	alpha	agonist	8517.8
CPD000499584	alpha	agonist	14606.7
CPD000466276	alpha	agonist	13964.4
CPD001370753	alpha	agonist	12695.6
CPD000058355	alpha	agonist	12160.0
CPD000469209	alpha	agonist	11453.3
CPD000058803	alpha	agonist	10846.7
CPD001906782	alpha	antagonist	11860.0
CPD000059133	alpha	antagonist	10914.4
CPD000059045	alpha	antagonist	10588.9
CPD000058180	alpha	antagonist	10424.4
CPD000058422	alpha	antagonist	9828.9
CPD000058520	alpha	antagonist	9760.0
CPD000058525	alpha	antagonist	14412.2
CPD000466362	alpha	antagonist	14320.0
CPD000466346	alpha	antagonist	13822.2
CPD000449301	alpha	antagonist	12813.3
CPD000466340	alpha	antagonist	12324.4
CPD000097306	alpha	antagonist	11308.9
CPD000466275	alpha	antagonist	10780.0
CPD000449268	alpha	antagonist	10511.1
CPD000058309	alpha	antagonist	10148.9
CPD001819784	alpha	antagonist	12466.7
CPD000471623	alpha	antagonist	9591.1
CPD000469190	alpha	antagonist	14555.6
CPD000449302	alpha	antagonist	14146.7
CPD000466347	alpha	antagonist	13660.0
CPD000550486	alpha	antagonist	13222.2
CPD000149600	alpha	antagonist	11915.6
CPD000058365	alpha	antagonist	10082.2
CPD000857209	alpha, beta	agonist	81826.7
CPD000058383	alpha, beta	agonist	33662.2
CPD000059081	alpha, beta	agonist	12310.0
CPD000058463	alpha, beta	antagonist	15577.8
CPD000466345	alpha, beta	antagonist	9731.1
CPD000449280	alpha, beta	antagonist	13964.4
CPD000058296	alpha, beta	antagonist	9591.1

CPD000058267	beta	agonist	81826.7
CPD000058729	beta	agonist	45628.9
CPD001453712	beta	agonist	45628.9
CPD000471620	beta	agonist	81826.7
CPD000466295	beta	agonist	81826.7
CPD001496939	beta	agonist	39806.7
CPD000058513	beta	agonist	33662.2
CPD000058420	beta	antagonist	33662.2
CPD000058800	beta	antagonist	16197.8
CPD000059167	beta	antagonist	14320.0
CPD000059120	beta	antagonist	13964.4
CPD000326828	beta	antagonist	13222.2
CPD000058388	beta	antagonist	13148.9
CPD000036768	beta	antagonist	12047.8
CPD000471619	beta	antagonist	10846.7
CPD000058975	beta	antagonist	10780.0
CPD000469141	beta	antagonist	10257.8
CPD001453705	beta	antagonsit	11308.9

Table S4. mRNA seq data for GPCRs and related genes

	TPM (transcripts per million)			TPM (transcripts per million)	
**Gene name**	**HEK293T**	**HeLa**	**Gene name**	**C2C12**	**Neuro2A**
**ACKR1**	NA	NA	**Ackr1**	NA	0.411135
**ACKR2**	0.0671921	0.00611861	**Ackr2**	0.0290849	0.0274002
**ACKR3**	NA	10.7309	**Ackr3**	10.5631	0.0218309
**ACKR4**	2.40158	1.43155	**Ackr4**	4.28392	0.273759
**ADCYAP1R1**	0.412282	0.0204383	**Adcyap1r1**	NA	10.1754
**ADGRA1**	0.014162	0.00209562	**Adgra1**	0.0280815	0.349205
**ADGRA2**	1.06185	1.11038	**Adgra2**	17.5953	3.67736
**ADGRA3**	1.52622	1.54665	**Adgra3**	4.41482	1.44618
**ADGRB1**	0.0186445	0.276368	**Adgrb1**	NA	0.139848
**ADGRB2**	0.99388	2.11289	**Adgrb2**	0.205729	0.369166
**ADGRB3**	0.55272	0.000928321	**Adgrb3**	NA	0.0175584
**ADGRD1**	0.00229335	0.0678718	**Adgrd1**	0.205819	4.37884
**ADGRD2**	0.00678628	0.569381	**Adgre1**	0.0296354	0.0217767
**ADGRE1**	0.00116175	0.00722024	**Adgre5**	20.6852	0.871518
**ADGRE2**	0.0053239	6.33217	**Adgrf1**	NA	0.00896804
**ADGRE3**	1.52864	9.58569	**Adgrf2**	0.0428473	0.292145
**ADGRE4P**	0.00135432	NA	**Adgrf3**	0.170553	0.0226834
**ADGRE5**	0.333102	16.6925	**Adgrf4**	NA	0.189065
**ADGRF1**	NA	0.00570313	**Adgrf5**	0.0398179	0.0858076
**ADGRF2**	0.00742829	NA	**Adgrg1**	4.8713	0.190748
**ADGRF3**	0.386397	0.331062	**Adgrg2**	0.119437	0.0467386
**ADGRF4**	0.00509844	0.0475298	**Adgrg3**	1.90625	0.134055
**ADGRF5**	0.00119993	0.0031073	**Adgrg4**	0.00656975	0.00530503
**ADGRG1**	0.00111871	4.58533	**Adgrg5**	0.214064	0.0142352
**ADGRG2**	0.161309	0.208143	**Adgrg6**	4.31215	3.71776
**ADGRG3**	NA	0.241309	**Adgrg7**	NA	0.0166323
**ADGRG4**	0.00318041	0.00164717	**Adgrl1**	11.9194	17.6022
**ADGRG5**	0.00125308	0.0986458	**Adgrl2**	5.19926	1.72021
**ADGRG6**	0.37275	0.133109	**Adgrl3**	0.298114	0.722868
**ADGRG7**	0.00572431	0.00370586	**Adgrl4**	0.0264586	0.0123546
**ADGRL1**	NA	NA	**Adgrv1**	0.00687546	0.0687879
**ADGRL1**	1.9325	1.72728	**Adora1**	14.243	0.173259
**ADGRL2**	0.00877483	0.00565755	**Adora2a**	0.406292	8.11576
**ADGRL3**	0.61066	0.000653147	**Adora2b**	5.09656	NA
**ADGRL4**	0.00125292	0.0629437	**Adora3**	NA	0.0292967
**ADGRV1**	0.776475	0.0175391	**Adra1a**	0.00181462	0.00923136
**ADORA1**	0.0640502	0.0514172	**Adra1b**	0.0915609	0.010461
**ADORA2A**	0.602473	0.759269	**Adra1d**	2.88152	0.0508989
**ADORA2B**	1.27165	1.93039	**Adra2a**	NA	0.31737
**ADORA3**	NA	NA	**Adra2b**	NA	1.47609
**ADRA1A**	0.0128956	NA	**Adra2c**	NA	0.0809359
**ADRA1B**	0.0142333	0.159227	**Adrb1**	0.0677892	0.0447038
**ADRA1D**	0.00643274	0.0666319	**Adrb2**	33.8209	NA
**ADRA2A**	0.0633666	NA	**Adrb3**	0.173753	0.0140304
**ADRA2B**	0.0498781	0.137773	**Agtr2**	NA	0.0301692
**ADRA2C**	5.97859	5.17038	**Aplnr**	0.666774	0.0565337
**ADRB1**	0.465069	1.36141	**Avpr1a**	NA	0.0281413
**ADRB2**	53.3648	2.82853	**Avpr1b**	NA	0.114702

**ADRB3**	NA	NA	**Avpr2**	0.217559	0.471402
**AGTR1**	0.134517	0.00985204	**Bdkrb1**	4.73498	0.118952
**AGTR2**	NA	0.030088	**Bdkrb2**	1.98779	2.53243
**APLNR**	NA	0.191354	**Brs3**	NA	NA
**AVPR1A**	0.0800601	NA	**C3ar1**	0.478302	0.015449
**AVPR1B**	0.0228353	NA	**C5ar1**	0.0481794	0.0980394
**AVPR2**	0.0397647	2.64984	**C5ar2**	0.0128861	NA
**BDKRB1**	NA	0.0798259	**Calcr**	0.00468096	0.0582096
**BDKRB2**	0.197633	0.538379	**Calcrl**	1.56514	0.0956025
**BRS3**	0.147463	NA	**Casr**	NA	0.00611045
**C3AR1**	0.0193726	0.0401332	**Cckar**	NA	0.0140395
**C5AR1**	0.0441064	0.572074	**Cckbr**	0.0409537	0.0077163
**C5AR2**	0.0121643	5.84222	**Ccr1**	0.037546	NA
**CALCR**	0.00245398	NA	**Ccr10**	1.76387	3.61776
**CALCRL**	0.0761653	0.00119536	**Ccr2**	NA	0.042035
**CASR**	NA	0.00235828	**Ccr3**	NA	NA
**CCKAR**	NA	NA	**Ccr4**	0.00844599	0.17664
**CCKBR**	0.074805	NA	**Ccr5**	0.047146	0.0239842
**CCR1**	NA	NA	**Ccr6**	0.0116896	0.0991128
**CCR10**	0.53764	5.2779	**Ccr7**	0.0679948	0.134518
**CCR2**	NA	NA	**Ccr8**	0.0883064	NA
**CCR3**	0.00178801	0.0265463	**Ccr9**	1.58616	12.525
**CCR4**	NA	0.108204	**Ccrl2**	1.5039	0.252724
**CCR5**	NA	NA	**Celsr1**	0.0118511	0.024734
**CCR6**	0.0881316	0.02054	**Celsr2**	NA	1.93016
**CCR7**	NA	1.23996	**Celsr3**	0.101803	12.6988
**CCR8**	0.061883	0.0320499	**Chrm1**	0.0373716	0.225324
**CCR9**	0.0663445	0.0152714	**Chrm2**	0.000878779	0.00596071
**CCRL2**	NA	0.087313	**Chrm3**	0.00177855	0.196755
**CELSR1**	0.499838	2.56748	**Chrm4**	1.88405	15.5386
**CELSR2**	1.65504	14.6301	**Chrm5**	0.154246	0.0435935
**CELSR3**	2.55111	17.0353	**Cmklr1**	0.715827	0.0549255
**CHRM1**	NA	0.0145444	**Cnr1**	4.17697	17.3266
**CHRM2**	0.100533	NA	**Cnr2**	NA	0.039328
**CHRM3**	0.0216107	0.0973624	**Crhr1**	NA	0.486826
**CHRM4**	0.447351	1.64288	**Crhr2**	0.0114802	0.00162228
**CHRM5**	0.195597	0.0611029	**Cx3cr1**	6.74777	1.03732
**CMKLR1**	NA	NA	**Cxcr1**	NA	NA
**CNR1**	0.201771	NA	**Cxcr2**	NA	0.019208
**CNR2**	0.0100389	0.0118841	**Cxcr3**	NA	NA
**CRHR1**	NA	NA	**Cxcr4**	9.96798	NA
**CRHR1**	NA	NA	**Cxcr5**	0.288044	0.20178
**CRHR1**	0.0882128	NA	**Cxcr6**	0.839863	0.0923084
**CRHR2**	0.0395315	0.184925	**Cysltr1**	NA	NA
**CX3CR1**	0.0505316	NA	**Cysltr2**	0.0576947	0.275717
**CXCR1**	NA	NA	**Drd1**	NA	0.123292
**CXCR2**	NA	0.0425654	**Drd2**	0.00182558	0.00515951
**CXCR3**	0.0710128	NA	**Drd3**	0.00715863	0.0890204
**CXCR4**	4.20335	19.1741	**Drd4**	0.110626	0.140694
**CXCR5**	0.106131	23.1592	**Drd5**	NA	0.0442297
**CXCR6**	1.78908	0.154431	**Ednra**	0.843089	0.00794254
**CYSLTR1**	0.00218589	NA	**Ednrb**	NA	0.0468171

**CYSLTR2**	0.00213679	0.00885335	**F2r**	70.0968	41.9615
**DRD1**	NA	NA	**F2rl1**	NA	0.00516494
**DRD2**	0.0102281	NA	**F2rl2**	0.418957	17.5242
**DRD3**	0.00171103	NA	**F2rl3**	1.35992	0.663867
**DRD4**	NA	NA	**Ffar1**	NA	NA
**DRD4**	0.107242	1.38855	**Ffar2**	NA	NA
**DRD5**	NA	NA	**Ffar3**	NA	0.0376993
**EDNRA**	0.531609	1.98593	**Ffar4**	NA	0.158119
**EDNRB**	0.122824	0.00397576	**Fpr1**	0.0990521	NA
**F2R**	3.71295	5.43684	**Fpr2**	0.120724	0.0113731
**F2RL1**	3.50702	2.1796	**Fpr3**	0.0176537	0.353411
**F2RL2**	0.240159	NA	**Fshr**	0.00114331	0.0739958
**F2RL3**	NA	0.0705667	**Fzd1**	105.367	0.398605
**FFAR1**	NA	NA	**Fzd10**	0.0758892	0.021448
**FFAR2**	NA	NA	**Fzd2**	57.4348	0.837309
**FFAR3**	NA	NA	**Fzd3**	0.533685	2.26795
**FFAR4**	NA	0.351785	**Fzd4**	11.0258	4.82702
**FPR1**	0.0041704	0.0043198	**Fzd5**	39.3239	6.21095
**FPR2**	0.0132821	0.013758	**Fzd6**	4.64439	1.4546
**FPR3**	0.0396016	NA	**Fzd7**	53.3606	32.5613
**FSHR**	0.00223604	0.000992632	**Fzd8**	4.64462	0.0700094
**FZD1**	3.98434	2.89911	**Fzd9**	2.15124	0.425593
**FZD10**	1.42297	32.7759	**Gabbr1**	20.3612	3.95453
**FZD2**	4.03269	23.5639	**Gabbr2**	NA	0.0188241
**FZD3**	3.16524	0.552535	**Galr1**	NA	NA
**FZD4**	7.82272	12.8324	**Galr2**	0.698813	0.929413
**FZD5**	8.55875	5.40804	**Galr3**	0.221003	0.208202
**FZD6**	6.57078	5.44383	**Gcgr**	0.208311	0.0336419
**FZD7**	13.9492	7.43889	**Ghrhr**	NA	NA
**FZD8**	5.23009	6.82315	**Ghsr**	0.0185165	NA
**FZD9**	1.96702	6.68297	**Gipr**	0.489997	2.07726
**GABBR1**	NA	NA	**Glp1r**	0.563206	2.35901
**GABBR1**	NA	NA	**Glp2r**	0.328397	0.0902697
**GABBR1**	NA	NA	**Gna12**	5.04073	2.53181
**GABBR1**	NA	NA	**Gna13**	15.065	18.3693
**GABBR1**	NA	NA	**Gna14**	0.00845337	0.0314567
**GABBR1**	NA	NA	**Gna15**	0.180029	0.213061
**GABBR1**	0.47765	0.780732	**Gnai1**	0.0362626	0.0541713
**GABBR2**	0.0559262	0.0116465	**Gnai2**	147.998	12.1378
**GALR1**	NA	NA	**Gnal**	1.14441	0.293256
**GALR2**	0.0891546	0.138523	**Gnao1**	7.7029	3.62646
**GALR3**	0.0876185	0.0907574	**Gnaq**	1.65148	5.21112
**GCGR**	NA	NA	**Gnas**	88.98	48.5078
**GCGR**	0.0062215	4.46596	**Gnat1**	0.0470776	0.0532208
**GHRHR**	0.00450297	NA	**Gnat2**	1.75478	2.12892
**GHSR**	0.0356851	NA	**Gnat3**	0.00645812	0.0304202
**GIPR**	0.253695	5.20641	**Gnaz**	0.225656	1.45212
**GLP1R**	0.00289019	NA	**Gnrhr**	NA	NA
**GLP2R**	NA	7.13428	**Gpbar1**	NA	1.88044
**GNA12**	0.87518	3.39553	**Gper1**	11.4568	0.749527
**GNA13**	7.74662	7.70489	**Gpr1**	0.0348438	0.048144
**GNA14**	0.0103669	0.00989057	**Gpr101**	NA	0.0294948

**GNA15**	0.00443409	0.296244	**Gpr107**	7.54281	2.90739
**GNAI1**	0.37166	0.530017	**Gpr119**	0.0379446	NA
**GNAI2**	4.25137	30.8026	**Gpr12**	NA	3.55643
**GNAL**	1.0252	1.548	**Gpr132**	0.0284213	NA
**GNAO1**	0.0529181	1.52942	**Gpr135**	0.782983	1.6818
**GNAQ**	1.19176	1.04051	**Gpr137**	22.4674	15.4727
**GNAS**	20.9845	45.9669	**Gpr139**	0.0112475	0.00317879
**GNAT1**	NA	0.282336	**Gpr141**	0.0992168	0.0177593
**GNAT2**	2.02671	1.6222	**Gpr142**	0.268018	0.0534692
**GNAT3**	0.0046004	NA	**Gpr143**	0.00922846	0.0234735
**GNAZ**	2.29504	0.754277	**Gpr146**	5.65246	1.54911
**GNRHR**	1.05672	0.784998	**Gpr149**	0.194552	0.00549848
**GPBAR1**	0.253343	0.0728942	**Gpr15**	0.301147	NA
**GPER1**	0.3388	7.21614	**Gpr150**	NA	0.0324818
**GPR1**	NA	NA	**Gpr151**	0.347968	0.118012
**GPR1**	0.0388267	0.210026	**Gpr152**	0.0414242	0.0117074
**GPR101**	NA	NA	**Gpr153**	5.56305	2.2093
**GPR107**	3.32241	8.076	**Gpr156**	0.0731507	0.0589593
**GPR119**	NA	NA	**Gpr157**	1.6874	0.0150204
**GPR12**	0.0329961	NA	**Gpr158**	0.00319615	0.051639
**GPR132**	0.00765682	0.21414	**Gpr160**	0.233226	0.0219717
**GPR135**	0.908525	2.66024	**Gpr161**	2.98769	0.434579
**GPR137**	3.14219	11.5587	**Gpr162**	3.27221	5.07067
**GPR139**	NA	NA	**Gpr17**	0.705746	0.440911
**GPR141**	0.00245811	0.0169744	**Gpr171**	NA	NA
**GPR142**	NA	NA	**Gpr173**	4.22904	2.40988
**GPR143**	0.006049	0.110694	**Gpr174**	0.012063	0.018751
**GPR146**	0.492734	3.53377	**Gpr176**	2.09097	0.0709292
**GPR148**	NA	NA	**Gpr179**	0.0494045	0.230387
**GPR149**	0.0135483	0.00534614	**Gpr18**	0.190603	0.0769552
**GPR15**	NA	NA	**Gpr182**	1.74314	0.563028
**GPR150**	NA	NA	**Gpr183**	0.0195049	0.0551254
**GPR151**	0.338292	NA	**Gpr19**	1.89041	2.93141
**GPR152**	NA	NA	**Gpr20**	0.0382328	0.0756382
**GPR153**	1.80603	9.25179	**Gpr21**	0.461872	0.163169
**GPR156**	0.0862185	0.951201	**Gpr22**	NA	5.34534
**GPR157**	1.21628	3.13415	**Gpr25**	NA	NA
**GPR158**	0.00460052	0.0735649	**Gpr26**	NA	0.142176
**GPR160**	1.99396	1.1079	**Gpr27**	NA	0.233521
**GPR161**	2.0774	0.900151	**Gpr3**	0.115721	5.29827
**GPR162**	0.802255	0.404062	**Gpr33**	NA	NA
**GPR17**	0.0816187	4.48076	**Gpr34**	0.140616	0.0953788
**GPR171**	0.981371	NA	**Gpr35**	0.32976	0.0706341
**GPR173**	0.00386149	0.553976	**Gpr37**	0.029773	0.0476824
**GPR174**	NA	NA	**Gpr37l1**	0.0331149	0.393079
**GPR176**	0.74089	1.01152	**Gpr39**	0.329491	0.0123926
**GPR179**	NA	NA	**Gpr4**	0.264931	0.155741
**GPR179**	0.0760753	0.0656672	**Gpr45**	0.0029865	0.098754
**GPR18**	1.2061	0.162953	**Gpr50**	NA	0.0124675
**GPR182**	0.233	0.273526	**Gpr52**	0.602663	0.0425816
**GPR183**	1.00816	0.445425	**Gpr55**	NA	0.00921631
**GPR19**	0.186169	0.151387	**Gpr6**	NA	0.120755

**GPR20**	NA	NA	**Gpr61**	0.18793	0.0743587
**GPR20**	0.0454468	0.841464	**Gpr62**	6.8615	2.18603
**GPR21**	0.778702	0.0467594	**Gpr63**	0.820654	0.00651047
**GPR22**	NA	NA	**Gpr65**	0.0307915	0.0261071
**GPR22**	1.16025	0.314493	**Gpr68**	1.43209	1.90206
**GPR25**	NA	NA	**Gpr75**	0.206221	0.566174
**GPR26**	NA	NA	**Gpr82**	0.243755	0.0688908
**GPR27**	15.4206	NA	**Gpr83**	NA	0.0131769
**GPR3**	0.327168	7.63495	**Gpr84**	0.103717	0.0586257
**GPR31**	NA	NA	**Gpr85**	0.764426	7.23112
**GPR32**	0.0484238	NA	**Gpr87**	NA	NA
**GPR33**	NA	NA	**Gpr88**	0.320311	0.0362109
**GPR34**	0.355244	0.298976	**Gprc5a**	6.95571	0.168239
**GPR35**	0.123708	0.211924	**Gprc5b**	27.9859	0.213647
**GPR37**	0.224392	13.1323	**Gprc5c**	2.46456	0.0389673
**GPR37L1**	0.207212	0.667753	**Gprc5d**	0.381205	0.0510334
**GPR39**	0.308081	0.137952	**Gprc6a**	NA	0.00837863
**GPR4**	0.00987224	0.132937	**Grm1**	0.0230273	0.141946
**GPR42**	NA	NA	**Grm2**	NA	0.0751374
**GPR45**	0.142492	0.368992	**Grm3**	0.00205851	0.0113447
**GPR50**	0.151044	0.0260759	**Grm4**	0.00249088	0.00915174
**GPR52**	1.20414	0.0804693	**Grm5**	0.00470225	0.0322748
**GPR55**	NA	0.117239	**Grm6**	NA	NA
**GPR6**	NA	NA	**Grm7**	0.023148	0.010059
**GPR61**	0.84237	0.372883	**Grm8**	0.00157724	0.0286909
**GPR62**	0.0579989	0.36046	**Grpr**	0.0999686	0.00584553
**GPR63**	2.46491	0.0205549	**Hcar1**	NA	0.0848779
**GPR65**	NA	NA	**Hcar2**	0.127793	0.0722342
**GPR68**	0.0143615	0.119008	**Hcrtr1**	0.0269758	0.0609918
**GPR75**	1.41955	3.72562	**Hcrtr2**	0.00251732	0.00853743
**GPR78**	0.387604	0.5235	**Hrh1**	0.0606768	0.200884
**GPR82**	0.330486	0.117674	**Hrh2**	NA	0.00949414
**GPR83**	0.132567	0.0580952	**Hrh3**	NA	1.94244
**GPR84**	NA	0.591959	**Hrh4**	NA	0.0350877
**GPR85**	0.598368	0.026948	**Htr1a**	NA	0.139909
**GPR87**	0.194607	0.0167982	**Htr1b**	1.91504	0.0251737
**GPR88**	0.0791267	NA	**Htr1d**	0.0590754	0.016696
**GPRC5A**	0.519478	179.4	**Htr1f**	0.0142979	0.0392545
**GPRC5B**	1.24038	0.557113	**Htr2a**	0.0808302	0.0111684
**GPRC5C**	0.414969	2.81886	**Htr2b**	1.00028	0.425398
**GPRC5D**	0.0324188	10.9975	**Htr2c**	0.135525	0.00682912
**GPRC6A**	0.0149476	NA	**Htr4**	0.0100159	0.0105141
**GRM1**	0.00824415	0.0080737	**Htr5a**	NA	0.0161606
**GRM2**	0.0691882	0.00551283	**Htr6**	0.0255797	0.583173
**GRM3**	0.108177	0.0483928	**Htr7**	0.0112366	0.0162314
**GRM4**	0.000897208	0.136614	**Kiss1r**	3.09288	1.57736
**GRM5**	0.00186089	0.000680313	**Lgr4**	3.70045	0.466818
**GRM6**	NA	NA	**Lgr5**	0.00897068	0.785441
**GRM7**	0.00120176	0.00746885	**Lgr6**	0.181983	0.00514326
**GRM8**	0.00588472	0.00265703	**Lhcgr**	NA	0.00830822
**GRPR**	0.00820589	0.495117	**Lpar1**	3.06418	0.0117504
**HCAR1**	0.051541	NA	**Lpar2**	1.3222	4.17079

**HCAR2**	NA	7.42854	**Lpar3**	0.308186	0.00106872
**HCAR3**	0.119552	6.25367	**Lpar4**	15.0574	0.0367537
**HCRTR1**	13.6587	7.28215	**Lpar5**	NA	0.349656
**HCRTR2**	0.00104648	NA	**Lpar6**	5.77777	3.1755
**HRH1**	0.00680265	1.98657	**Ltb4r2**	NA	0.0751345
**HRH2**	0.026805	0.00120718	**Mas1**	0.337176	0.0669631
**HRH3**	NA	0.203435	**Mc1r**	0.0677954	0.36405
**HRH4**	0.0540567	NA	**Mc2r**	0.187065	1.68558
**HTR1A**	NA	NA	**Mc3r**	NA	NA
**HTR1B**	NA	NA	**Mc4r**	2.0678	0.0248684
**HTR1D**	0.476859	8.06024	**Mc5r**	0.203372	0.0287388
**HTR1E**	NA	0.000804164	**Mchr1**	1.31646	0.160891
**HTR1F**	0.09785	NA	**Mrgprd**	NA	NA
**HTR2A**	0.0103156	NA	**Mrgpre**	11.7735	1.28328
**HTR2B**	1.07454	0.162791	**Mrgprf**	14.5498	0.391248
**HTR2C**	0.00113103	0.00488147	**Mrgprg**	NA	NA
**HTR4**	0.00271656	0.00759749	**Mrgprx1**	NA	0.0105185
**HTR5A**	NA	NA	**Mrgprx2**	0.0774361	12.2995
**HTR6**	0.087829	0.0632873	**Mtnr1a**	NA	0.00719732
**HTR7**	0.0644815	0.0179197	**Mtnr1b**	NA	0.0236863
**KISS1R**	0.0991392	NA	**Nmbr**	1.50229	49.4062
**LGR4**	3.37246	15.8887	**Nmur1**	0.0185323	0.0209506
**LGR5**	0.209288	0.00130332	**Nmur2**	NA	NA
**LGR6**	0.00878684	0.273049	**Npbwr1**	NA	0.0188803
**LHCGR**	0.0115847	0.00276917	**Npffr1**	0.0111858	0.0358288
**LPAR1**	0.30614	0.00154123	**Npffr2**	NA	0.0123785
**LPAR2**	0.163059	2.24396	**Npsr1**	0.00677573	0.00414911
**LPAR3**	1.02485	NA	**Npy1r**	0.924803	0.0435617
**LPAR4**	NA	NA	**Npy2r**	NA	0.136509
**LPAR5**	0.0071762	2.32662	**Npy4r**	0.203163	NA
**LPAR6**	0.454705	0.349783	**Npy5r**	0.0302107	0.0341529
**LTB4R**	NA	NA	**Npy6r**	NA	NA
**LTB4R**	4.66447	13.2965	**Ntsr1**	0.00274014	0.00154885
**LTB4R2**	NA	NA	**Ntsr2**	0.108081	0.336006
**LTB4R2**	19.3295	23.0131	**Opn1mw**	0.0211735	8.35383
**MAS1**	NA	0.00721669	**Opn1sw**	0.742135	0.0886244
**MAS1L**	NA	NA	**Opn3**	3.99339	1.40762
**MAS1L**	NA	NA	**Opn4**	0.103576	NA
**MAS1L**	NA	NA	**Opn5**	NA	0.00765818
**MAS1L**	NA	NA	**Oprd1**	0.0255691	0.251892
**MAS1L**	NA	NA	**Oprk1**	NA	0.0315662
**MAS1L**	NA	NA	**Oprl1**	0.0413132	2.05498
**MAS1L**	NA	NA	**Oprm1**	0.00264547	0.016698
**MAS1L**	NA	NA	**Oxgr1**	NA	NA
**MC1R**	1.04787	10.2072	**Oxtr**	0.0455027	0.0600138
**MC2R**	NA	NA	**P2ry1**	NA	0.0225294
**MC3R**	NA	NA	**P2ry10**	0.0149633	0.0422896
**MC4R**	0.143407	NA	**P2ry12**	0.00264635	0.0254292
**MC5R**	NA	NA	**P2ry13**	NA	0.163137
**MCHR1**	0.0302336	NA	**P2ry14**	0.0248104	0.00175299
**MCHR2**	0.0056801	0.000840513	**P2ry2**	3.9949	0.359243
**MLNR**	0.150612	0.124806	**P2ry4**	0.0522985	NA

**MRGPRD**	NA	0.263566	**P2ry6**	0.103266	0.176995
**MRGPRE**	NA	0.0344619	**Prlhr**	NA	0.0443141
**MRGPRF**	NA	0.899388	**Prokr1**	0.629131	0.69402
**MRGPRG**	NA	NA	**Prokr2**	NA	0.0701224
**MRGPRX1**	NA	NA	**Ptafr**	0.119233	0.0299537
**MRGPRX2**	NA	NA	**Ptgdr**	NA	NA
**MRGPRX3**	0.0105186	0.00726363	**Ptgdr2**	1.08297	0.481899
**MRGPRX4**	NA	NA	**Ptger1**	39.192	10.0268
**MTNR1A**	0.0813236	NA	**Ptger2**	0.181515	NA
**MTNR1B**	0.0119583	0.0206445	**Ptger3**	0.24821	0.0070591
**NMBR**	0.598969	0.515978	**Ptger4**	8.76749	2.31443
**NMUR1**	0.118515	NA	**Ptgfr**	1.55615	0.0152533
**NMUR2**	0.0293856	0.00608767	**Ptgir**	16.0573	39.3517
**NPBWR1**	0.0944758	0.12582	**Pth1r**	0.167307	0.111258
**NPBWR2**	NA	NA	**Pth2r**	0.0207088	0.0565769
**NPBWR2**	NA	NA	**Qrfpr**	0.0172516	0.0162523
**NPFFR1**	NA	0.024297	**Rho**	0.743877	0.176599
**NPFFR2**	0.0269554	0.0136868	**Rxfp1**	0.00256196	0.0101369
**NPSR1**	0.00139586	0.000289172	**Rxfp2**	NA	0.00878048
**NPY1R**	0.176656	NA	**Rxfp3**	0.144234	0.0163055
**NPY2R**	0.116451	NA	**Rxfp4**	0.891469	0.111978
**NPY4R**	0.037932	14.8781	**S1pr1**	46.7234	0.600914
**NPY5R**	0.0382008	NA	**S1pr2**	12.391	2.64884
**NPY6R**	0.179997	0.173128	**S1pr3**	11.6448	0.0443078
**NTSR1**	0.00683586	2.96093	**S1pr4**	NA	0.116858
**NTSR2**	0.00511315	0.0211853	**S1pr5**	0.156045	0.113405
**OPN1LW**	0.0207741	NA	**Sctr**	0.0892628	0.980804
**OPN1MW**	0.0215371	NA	**Smo**	14.9102	0.0889417
**OPN1SW**	10.4651	18.0731	**Sstr1**	NA	0.0260778
**OPN3**	12.3259	13.9307	**Sstr2**	NA	1.02156
**OPN4**	NA	0.0103481	**Sstr3**	0.0803073	0.508405
**OPN5**	NA	NA	**Sstr4**	NA	0.195803
**OPRD1**	0.226568	0.0150715	**Sstr5**	NA	NA
**OPRK1**	NA	NA	**Sucnr1**	NA	NA
**OPRL1**	NA	NA	**Taar1**	NA	NA
**OPRL1**	0.116552	0.238359	**Taar2**	NA	NA
**OPRM1**	0.00935894	0.00403926	**Taar5**	NA	NA
**OR1A1**	NA	NA	**Taar6**	NA	NA
**OR1G1**	NA	NA	**Taar9**	NA	NA
**OR2T11**	NA	NA	**Tacr1**	NA	0.00441818
**OR51E1**	NA	NA	**Tacr2**	0.0272973	0.072005
**OXER1**	0.207018	NA	**Tacr3**	0.0046724	0.02509
**OXGR1**	0.0477306	NA	**Tas1r1**	0.694434	0.588788
**OXTR**	0.479552	1.0332	**Tas1r2**	0.0515582	NA
**P2RY1**	19.3826	NA	**Tas1r3**	1.95746	0.476622
**P2RY10**	NA	NA	**Tbxa2r**	0.268003	0.205591
**P2RY11**	27.7526	32.5348	**Tpra1**	15.236	4.63404
**P2RY12**	0.98374	NA	**Trhr**	0.0248016	0.0122666
**P2RY13**	0.189777	NA	**Tshr**	0.0246273	0.0236226
**P2RY14**	0.442191	0.00287468	**Uts2r**	0.0724133	0.0409313
**P2RY2**	0.0865239	2.76515	**Vipr1**	0.024469	0.0345775
**P2RY4**	0.11558	0.0399067	**Vipr2**	0.16374	0.0122049

**P2RY6**	NA	2.84855	**Xcr1**	0.0126938	NA
**P2RY8**	0.00164734	0.0307143	**Cdc42**	43.8568	21.657
**PRLHR**	NA	NA	**Rac1**	55.5175	23.4372
**PROKR1**	0.0376915	NA	**Rhoa**	46.2479	12.3628
**PROKR2**	0.0249188	NA	**Rhog**	9.19874	1.53146
**PTAFR**	0.110363	0.187806			
**PTGDR**	NA	NA			
**PTGDR2**	3.83296	5.86027			
**PTGER1**	0.0212703	0.308452			
**PTGER2**	0.133269	0.035624			
**PTGER3**	0.0154052	0.00293089			
**PTGER4**	0.182291	4.79882			
**PTGFR**	1.00315	0.0142452			
**PTGIR**	NA	0.0275427			
**PTH1R**	0.0683325	0.0439327			
**PTH2R**	0.00248387	0.00192964			
**QRFPR**	0.219221	NA			
**RHO**	0.0091634	NA			
**RXFP1**	0.00272656	0.0826561			
**RXFP2**	0.0019244	NA			
**RXFP3**	NA	NA			
**RXFP3**	NA	NA			
**RXFP4**	0.296654	0.965742			
**S1PR1**	1.12151	2.05386			
**S1PR2**	1.48363	1.69754			
**S1PR3**	1.08342	2.00844			
**S1PR4**	0.0153875	1.41057			
**S1PR5**	0.296001	NA			
**SCTR**	0.0287622	0.00372408			
**SMO**	6.11217	NA			
**SSTR1**	0.0356989	9.25679			
**SSTR2**	1.50664	0.109517			
**SSTR3**	NA	0.0154474			
**SSTR4**	NA	NA			
**SSTR5**	NA	3.13115			
**SUCNR1**	NA	NA			
**TAAR1**	NA	NA			
**TAAR2**	NA	NA			
**TAAR3P**	NA	NA			
**TAAR5**	NA	NA			
**TAAR6**	NA	NA			
**TAAR8**	NA	NA			
**TAAR9**	NA	NA			
**TACR1**	0.169425	0.218328			
**TACR2**	0.0236055	0.948704			
**TACR3**	0.00275241	0.00997854			
**TAS1R1**	0.0502677	0.0442583			
**TAS1R2**	0.00611928	0.025354			
**TAS1R3**	0.609168	2.96566			
**TAS2R1**	0.00147393	0.0183208			
**TAS2R10**	NA	NA			
**TAS2R10**	NA	NA			

**TAS2R10**	1.2974	1.83257
**TAS2R13**	NA	NA
**TAS2R13**	NA	NA
**TAS2R13**	1.31383	0.466594
**TAS2R14**	NA	NA
**TAS2R14**	NA	NA
**TAS2R14**	1.43412	0.57028
**TAS2R16**	NA	NA
**TAS2R19**	NA	NA
**TAS2R19**	NA	NA
**TAS2R19**	3.6183	3.9385
**TAS2R20**	NA	NA
**TAS2R20**	NA	NA
**TAS2R20**	3.60422	3.64116
**TAS2R3**	1.56276	0.404685
**TAS2R30**	NA	NA
**TAS2R30**	NA	NA
**TAS2R30**	2.76834	0.113191
**TAS2R31**	NA	NA
**TAS2R31**	NA	NA
**TAS2R31**	2.40744	1.1845
**TAS2R38**	0.0537618	NA
**TAS2R39**	0.120845	NA
**TAS2R4**	2.69409	0.202953
**TAS2R40**	0.294582	NA
**TAS2R41**	NA	NA
**TAS2R41**	NA	NA
**TAS2R42**	NA	NA
**TAS2R42**	0.260105	NA
**TAS2R43**	NA	NA
**TAS2R43**	NA	NA
**TAS2R43**	NA	NA
**TAS2R45**	NA	NA
**TAS2R46**	NA	NA
**TAS2R46**	NA	NA
**TAS2R46**	2.24655	0.342211
**TAS2R5**	2.93635	0.497707
**TAS2R50**	NA	NA
**TAS2R50**	NA	NA
**TAS2R50**	1.84349	0.827465
**TAS2R60**	NA	NA
**TAS2R60**	NA	NA
**TAS2R7**	NA	NA
**TAS2R7**	NA	NA
**TAS2R7**	NA	NA
**TAS2R8**	NA	NA
**TAS2R8**	NA	NA
**TAS2R8**	0.197747	NA
**TAS2R9**	NA	NA
**TAS2R9**	NA	NA
**TAS2R9**	0.0571626	NA
**TBXA2R**	0.0397444	3.14422

**TPRA1**	3.62526	7.53851
**TRHR**	0.11594	0.0564074
**TSHR**	0.0234475	0.00532329
**UTS2R**	NA	NA
**VIPR1**	0.392057	0.520812
**VIPR2**	0.0189574	0.147819
**XCR1**	0.028664	NA
**CDC42**	18.1935	12.5121
**RAC1**	14.4102	40.8616
**RHOA**	31.575	29.2585
**RHOG**	1.69644	10.5251

Table S5. Primer Sequences

Primer Name	Corresponding cDNA Name	Sequence
CDH-ADRB2F	β2-AR	GTCGTGATCTAGAGCTAGCG CACC ATGGGGCAACCCGGGAACGGCAGC
CDH-ADRB2R	β2-AR	CCAGAGGTTGATTGTCGACGC TTACAGCAGTGAGTCATTTGTACTACAATTCCTCCCTTG
CIG-GNAQF	Gaq	TCGAGCTCAAGCTTCGCACC ATGACTCTGGAGTCCATCATGGCG
CIG-GNAQR	Gaq	TAGAAGCTTCTGCAGAT TTAGACCAGATTGTACTCCTTCAGGTTCAAC
Q_Q209LR	Gaq CA	CAGGCCCCCTACATCGACCATTCTG
Q_Q209LF	Gaq CA	TGGTCGATGTAGGGGGCCTGAGGTCAGAGAGAAGAAAATGGATACACTGC
CIG-GNASF	Gas	TCGAGCTCAAGCTTCGCACC ATGGGCTGCCTCGGGAACAGTAAG
CIG-GNASR	Gas	TAGAAGCTTCTGCAGAT TTAGAGCAGCTCGTACTGACGAAGGTG
S_Q227LR	Gas CA	TCATCGCGCAGGCCACCCACGTCAAACATGT
S_Q227LF	Gas CA	GACGTGGGTGGCCTGCGCGATGAACGCCGCAAG
CIG-GNAi2F	Gai	TCGAGCTCAAGCTTCGCACC ATGGGCTGCACCGTGAGCG
CIG-GNAi2R	Gai	TAGAAGCTTCTGCAGAT TCAGAAGAGGCCGCAGTCCTTCAGGTTG
i2_Q205LF	Gai CA	GTGGGTGGTCTGCGGTCTGAGCGG
i2_Q205LR	Gai CA	CCGCTCAGACCGCAGACCACCCAC
CIG-EPAC2F	EPAC	TCGAGCTCAAGCTTCGCACC ATGGTCGCTGCGCACGCTG
CIG-EPAC2R	EPAC	TAGAAGCTTCTGCAGAT CTATGGTCGACGAGGCTCTAATCTGTG
R432KF	EPAC DN	CTAGTGAATGATGCCCCAAAGGCTGCCTCTATCGTCTTA
R432KR	EPAC DN	TAAGACGATAGAGGCAGCCTTTGGGGCATCATTCACTAG
CIG-RAP1AS17NF	RAP1A DN	TCGAGCTCAAGCTTCGCACC ATGCGTGAGTACAAGCTAGTGGTCCTTGGTTCAGGAGGCGTTGGGAAGAATGCTCTGACAGTTCAGTTTG
CIG-RAP1AS17NR	RAP1A DN	TAGAAGCTTCTGCAGAT CTAGAGCAGCAGACATGATTTCTTTTTAGGCTTCTTCTTTTCC
